# Atrophy of diaphragm muscle visualized with ultrasound in mechanically ventilated patients

**DOI:** 10.1186/cc14304

**Published:** 2015-03-16

**Authors:** T Schepens, M Mergeay, W Verbrugghe, P Parizel, M Vercauteren, PG Jorens

**Affiliations:** 1 Antwerp University Hospital, Edegem, Belgium

## Introduction

Mechanical ventilation (MV) induces diaphragmatic muscle atrophy and contractile fibre dysfunction, the so-called ventilator-induced diaphragm dysfunction (VIDD). Although diaphragmatic atrophy can be assessed using ultrasound, the biggest trial in humans published so far included seven patients and only measuring the thickness at two moments during the disease process [1]. We aimed to assess the time course of diaphragm atrophy in a larger cohort of MV patients using ultrasound.

## Methods

**Figure 1:**
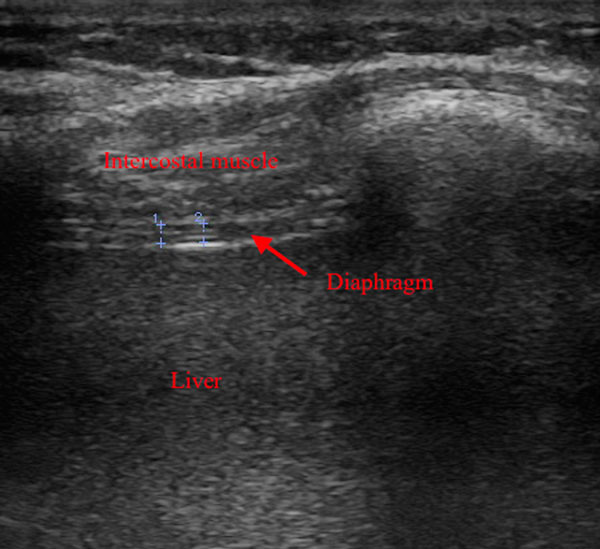
**Sample ultrasound recording**. Lines 1 and 2 are diaphragm thickness measurements.

A total of 54 patients from an adult ICU were included in this prospective single-centre cohort trial. Patients who needed <72 hours of MV or had been recently admitted to an ICU were excluded. Patients were ventilated in a controlled, assisted, and/or hybrid ventilation mode. The thickness of the diaphragm was assessed daily; the first recording was within 24 hours after the start of mechanical ventilation and we continued the measurements until the patients were extubated or tracheotomised. We measured the diaphragm at the zone of apposition, as described by McCool and colleagues [2] using a linear 13 MHz ultrasound probe. Figure 1 shows a sample measurement.

## Results

We were successfully able to record the diaphragm thickness in all included patients. Median time on the ventilator was 9 days (IQR 4 to 15 days). Mean baseline thickness was 1.9 mm (SD ±0.4 mm), and mean nadir was 1.3 mm (SD ±0.4 mm), corresponding with a mean change in thickness of 32% (SD ±18%). As early as after only 72 hours of MV, we already noted an average drop of diaphragm thickness of 20%, illustrating the rapid progression of the atrophy in VIDD.

## Conclusion

On average, diaphragm thickness decreased 32% in our cohort. The decrease occurred rapidly, with two-thirds of the maximal thinning already present after 72 hours of MV.
